# Approach direction and accuracy, but not response times, show spatial-numerical association in chicks

**DOI:** 10.1371/journal.pone.0257764

**Published:** 2021-09-30

**Authors:** Rosa Rugani, Lucia Regolin

**Affiliations:** Department of General Psychology, University of Padova, Padova, Italy; University of New England, Australia, AUSTRALIA

## Abstract

Chicks trained to identify a target item in a sagittally-oriented series of identical items show a higher accuracy for the target on the left, rather than that on the right, at test when the series was rotated by 90°. Such bias seems to be due to a right hemispheric dominance in visuospatial tasks. Up to now, the bias was highlighted by looking at accuracy, the measure mostly used in non-human studies to detect spatial numerical association, SNA. In the present study, processing by each hemisphere was assessed by scoring three variables: accuracy, response times and direction of approach. Domestic chicks were tested under monocular vision conditions, as in the avian brain input to each eye is mostly processed by the contralateral hemisphere. Four-day-old chicks learnt to peck at the 4^th^ element in a sagittal series of 10 identical elements. At test, when facing a series oriented fronto-parallel, birds confined their responses to the visible hemifield, with high accuracy for the 4^th^ element. The first element in the series was also highly selected, suggesting an anchoring strategy to start the proto-counting at one end of the series. In the left monocular condition, chicks approached the series starting from the left, and in the right monocular condition, they started from the right. Both hemispheres appear to exploit the same strategy, scanning the series from the most lateral element in the clear hemifield. Remarkably, there was no effect in the response times: equal latency was scored for correct or incorrect and for left vs. right responses. Overall, these data indicate that the measures implying a direction of choice, accuracy and direction of approach, and not velocity, i.e., response times, can highlight SNA in this paradigm. We discuss the relevance of the selected measures to unveil SNA.

## Introduction

In the 19^th^ century, Sir Francis Galton first outlined that humans represent numbers on a Mental Number Line, MNL, typically oriented from left-to-right. Along this MNL, small numbers are located on the left and large numbers on the right [1]. The seminal, and widely replicated, experimental demonstration of the MNL goes back to 1993, when Stanislas Dehaene and colleagues demonstrated that adults responded faster to small numbers on their left and to large numbers on their right: this was deemed the spatial-numerical association of response codes: SNARC effect [2]. Since this first evidence, a large body of literature has focused on replicating how number processing can modulate response times, depending on number magnitude and spatial displacement [3]. Other studies highlighted the MNL in humans by applying the selection of the responses [4]. Adult humans, asked to report random numbers within the interval 1–30, generated smaller numbers as facing leftward rather than rightward [5]. Likewise, participants watching leftward or downward gazes, produced smaller numbers than when watching rightward or upward gazes [6]. Also lateral arm turns–congruent body movements such as right-turns of both an arm and head–induced participants to generate smaller numbers when the movements were directed toward their left and bigger ones when movements were directed to their right [7]. In a dual task, which required estimating whether a number was smaller or bigger than five while moving a block in peripersonal space, adults while processing a smaller number placed a cube leftward—and rightward while processing larger numbers [8]. In executing an uncommon action, such as flicking a ball with the index finger toward one of two lateral goals upon detecting a visual stimulus, participants flicked leftward in response to small numbers and rightward when responding to large ones [9]. Overall, this evidence shows an intertwined interaction between numbers and action influence. Interestingly, this interaction is not confined to laboratories, but it occurs also in ordinary activities. Numerical magnitude influences directional decisions while walking. In a recent study, healthy adults were required to walk and produce random numbers as they made lateral turns. After the generation of small numbers, the participants turned left—and after the generation of larger numbers they turned right [10]. Thus, the association between numbers and space is not limited to the speed of action executions—instead it also affects action selection [9, 11, 12]. Nevertheless, an open question is whether an experimental paradigm designed to measures the SNA by action selection might assess it also by response times and vice-versa.

A much discussed aspect of the MNL concerns the origin of its orientation. Since the left-to-right orientation is diminished or even inverted in right to left reading cultures, it has been suggested MNL originates from culturally specific experiences [13, 14]. Yet, a developing number of evidence supports a phylogenetic origin of MNL [15]. Recent evidence from studying preschoolers [16–19], infants [20, 21] and newborns [22, 23] rules out a primary influence of verbal culture in MNL orientation. Eight-month-old infants presented with an array of dots shifted their attention toward their left or their right, depending on the array’s numerousness. When the number was small, such as two, they turned to their left—whenever it was large, such as nine, they turned rightward [20]. Three-day-old newborns habituated to an array of 12 items looked longer at a smaller number, four, when it was at their left and at a larger number, like 36, at their right side [23]. Newborns, once habituated with a sequence of sounds, when presented with two quantitatively identical visual arrays looked longer at the left one when they depicted a smaller quantity and at the right one when they depicted a larger quantity [22].

A left-to-right oriented proto-counting has been found in animals. Day-old domestic chicks, adult Clark’s nutcrackers [24] and adult monkeys [25] learnt to identify a target item in a sagittal sequence of identical items. When they faced an identical sequence but rotated by 90°, they mostly selected the target item from the left end, ignoring the one from the right end. Day-old birds also show an association of large numerousness with their right space. Chicks reared with a set of identical objects saw a set of objects disappearing behind a screen and a second set disappearing behind another screen. Chicks performed better when the larger set was on their right side, suggesting that bigger numbers are preferentially associated with the right space [26]. Employing an alternative paradigm, designed to investigate spatial choices, new-born chicks learnt to circumnavigate a panel depicting a numerical target (5 dots). At test, chicks faced two identical numerousness, one on their left and one on their right side. When faced with two smaller numerousness (2vs.2) birds showed a left choice—when faced with a bigger numerousness (8vs.8), they showed a right choice, and they showed no preference in the control test 5vs.5 [27, 28]. SNA has been mainly investigated in non-symbolic subjects considering direction of choices and only a few studies used the response times. Adult chimpanzees learnt to touch in ascendant order, Arabic numerals 1 through 9 presented in random positions on a screen. During testing, only the two extreme numerals, 1 and 9, were horizontally displayed on the screen. A numeral on their left and the other numeral on their right. Chimpanzees responded faster to the Left-Right condition (1_9) than the Right-Left condition (9_1) [29]. Gorillas and orangutans showed a space-magnitude correspondence. They learnt to pick either the smaller or the larger quantity in a pair of dot arrays, displayed on a screen, depending on the experimental condition. Response times to the left and right displayed a spatial representation of magnitude. Yet, present in most apes, SNA was either left-to-right or right-to-left oriented, depending on the individual [30]. Likewise, adult blue jays (*Cyanocitta cristata*) and domestic pigeons (*Columba livia*) trained to select the smaller or the larger of two nonadjacent quantity arrays showed a robust tendency to spatially represent quantities [31]. Despite of its individual directionality, the presence of SNA in most of the subjects suggests that it is a widespread cognitive strategy. Idiosyncratic experiences, such as interactions with their caregivers, perhaps determine the individual orientation of the spatial numerical association in adult animals [30]. Interestingly, this evidence is based on birds’ accuracy, instead of response times, which were used with primates, since accuracy in pigeons demonstrated to parallel the patterns observed for response times in human and non-human primates [31–33]. The selection of the measure used to document the selection of spatial numerical association is a crucial point that not only allows to obtain valid data, but that it should also be taken into consideration, in paradigm design and in replication between different species.

Up to now, spatial numerical association, SNA, in animals has been mainly investigated by applying dichotomous left-right choices [27, 28, 34, 35]. Concerning the ordinal counting paradigm, the only previously used measure has been accuracy: the percentage of correct responses scored on either the left or right target element. Accuracy was used to study SNA in chicks [24, 34, 36–38], Clark nutcrackers [24] and rhesus monkeys [25].

The goal of the present study is to deepen our knowledge of spatial numerical association by analyzing three variables: accuracy, response times and direction of approach, in non-human animals. The innovative element here consists in assessing whether the new measures (response times and approach direction) allow for the detection of SNA within the same paradigm for which accuracy highlights SNA.

We used a paradigm previously employed to explore SNA in a spatial-ordinal task [24, 25, 34, 36–38]. Chicks learnt to select the fourth container in a series of identical, equidistant and sagittally displaced containers. Then they faced a sagittal test and a fronto-parallel test, which was conducted in two conditions of vision, left and right monocular. Monocular conditions were obtained by restricting the visual input to one eye using a simple and removable patch over the other eye [39, 40]. By testing the chicks monocularly, we determine the functioning of the one hemisphere which is in charge of processing most of the information entering the eye in use, which is the contralateral hemisphere [41–44]. This is due to the fact that the avian brain displays a virtually complete decussation of the fibers at the optic chiasm [45]; therefore, information entering one eye is projected to the opposite side of the brain and from here mainly to the ipsilateral hemisphere (for more detailed information on projections in chick brain, see [46]). Moreover the information is largely confined within the processing hemisphere as the avian brain having no *corpus callosum* [47] has no fast and effective inter-hemispheric communication [41–44]. There are some commissures in the bird brain, such as the anterior, the tectal, the hippocampal and posterior commissures but these are of lesser importance than the mammalian interhemispheric commissure [48–52]. Whenever a hemisphere is dominant for a specific cognitive process, the behavior of binocular chicks is identical to the one obtained in either monocular condition [53]. In an ordinal paradigm, equal to the one employed in this study, chicks that underwent at a fronto-parallel test in binocular and left monocular condition performed similarly [36]. Thus, we decided to test chicks in monocular conditions to disentangle the engagement of either hemisphere in determining the spatial bias. As in previous studies, we recorded accuracy as the numbers of pecks emitted at a target container. This measure is based on action selection: more specifically which container the chicks pecked during each trial. Further, we analyzed the chicks’ behavior through two additional variables. Direction of approach, i.e., the path taken while approaching the target, a new measure focused on action selection, and response times, aimed to unveil whether chicks’ speed was modulated by the direction of their responses. Since this paradigm was primarily designed to record action selection, we predicted a spatial bias would emerge for the Direction of approach, but not for the Responses times.

## Material and methods

### Ethical statement

Animal experimentation: The experiments complied with all applicable national and European laws concerning the use of animals in research and were approved by the Italian Ministry of Health (permit number: 32662 granted on 19/07/2011). All procedures employed in the experiments included in this study were examined and approved by the Ethical Committee of the University of Padova (*Comitato Etico di Ateneo per la Sperimentazione Animale—CEASA*) as well as by the Italian National Institute of Health (NIH).

### Subjects

Subjects were 24 male domestic chicks (*Gallus gallus*, broilers of a commercial hybrid), which were purchased from local commercial hatcheries each week (Agricola Berica, Montegalda, Vicenza, Italy). At their arrival, chicks were a few hours old and they were immediately caged in groups of two in standard metal cages (28 × 40 × 32 cm; width, depth, height) at a controlled humidity of 68 per cent at 28–31 Celsius. Chick starter crumbs and water were freely available. Three times a day, we also fed chicks with mealworms (*Tenebrio molitor* larvae), as these were used as reinforcement during training. Chicks were reared in these conditions from Monday morning at 11:00 to Wednesday morning at 10:00, when they were individually caged. On Wednesday evening at 8:00, the food available was measured to leave available a quantity that was sufficient up to Thursday morning around 8:00. On Thursday morning at 10:00, the chicks underwent pre-training. Two hours after their pre-training, each bird underwent training. Owing to yolk sac reserve, chicks are unmotivated to peck for food reward before the fourth day post-hatching, thus experimental procedures started on Thursday. Once training was over, they were singly caged overnight with a quantity of food that was sufficient until Friday morning at 8:00. Water was always freely available in their rearing cages. One hour after the end of training, each bird individually underwent a test. The interval between two consecutive tests was one hour. Immediately after the end of the tests, the chicks were caged in groups of two, with food and water freely available. A few hours later, all chicks were donated to local farmers.

Though the order of the monocular tests had been previously shown to not affect chicks’ performance in an ordinal task [34, 36], a group of chicks (n = 12) underwent the left fronto-parallel test as the first; and another group (n = 12) underwent the right fronto-parallel test as the first.

Sample size was planned using the formula for quantitative variables: *n = (2σ*^*2*^*)/(μ1–μ2)*^*2*^ *× f(α*,*β)*; with the following values: α = 0.05; β = 0.80; average = 30%; and SD = 18% [37]. In this formula, σ is the variance, μ1 and μ2 are the means of the two groups, and f(α,β) is a function of the type I error (α) and type II error (β). The sample size was evaluated according to the reduction principle in animal research and approved by the University Committee for animal welfare.

### Apparatus

**Fig 1:**
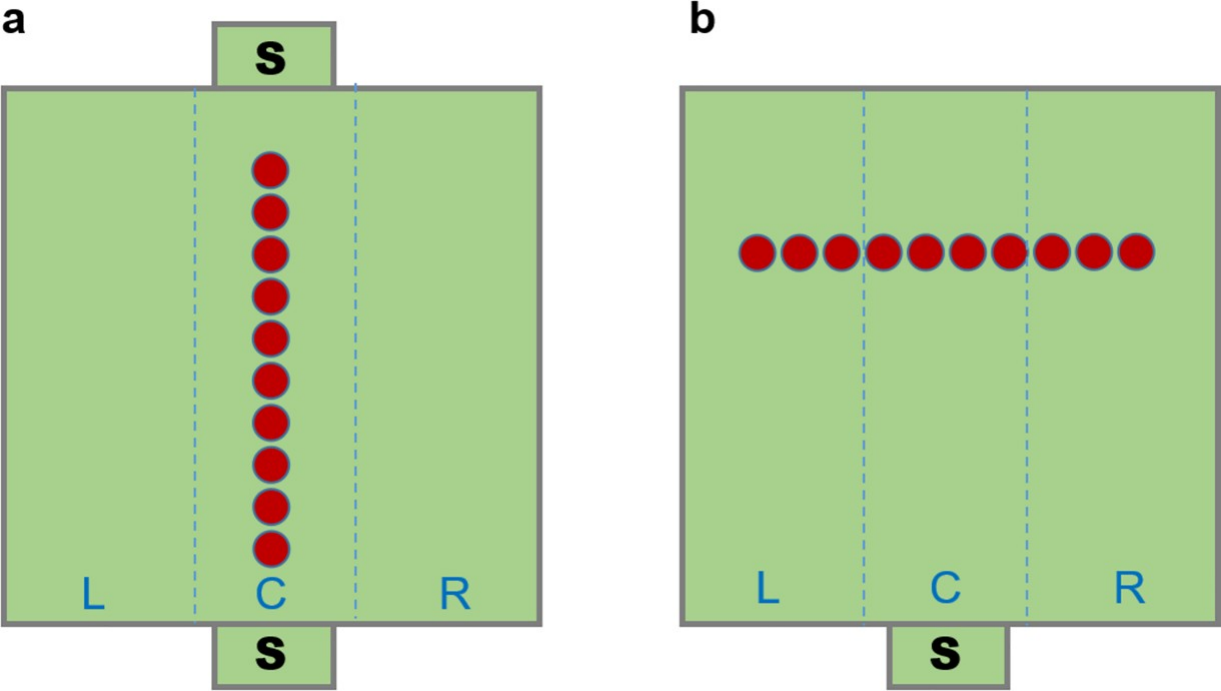
Schematic illustration of the apparatus and the series of elements. S represents the starting box. In light blue the three sectors used to evaluate the direction of approach to the series: L, left; C, central or R, right. (a) The position of the series during training, retraining and sagittal test, with two possible starting positions. (b) The displacement of the series for the fronto-parallel tests.

All experimental phases; pre-training, training, re-training, sagittal test and fronto-parallel tests took place in a dedicated room located nearby the rearing room. The room temperature was 25 degrees Celsius and the humidity was kept at 70 per cent. The experimental apparatus was a 106 cm squared plywood arena with 40 cm high side walls. The inner apparatus was lit by four 58W lamps, placed 148 cm above it. Two openings, 7.0 cm wide x 11.0 cm high, were halfway between two opposing walls. Each opening connected the arena with a starting box made of dark green polypropylene (7.0 x 11.0 x 11.5 cm, length, width and height), located outside of the apparatus and connected with it. The openings were usually closed by an 8 x 12 cm opaque plastic partition, which was lifted for a few seconds at the beginning of each trial to let the chicks enter the apparatus. The floor was covered by wood shavings. Ten identical elements (red plastic bottle caps, 3.2 cm x 0.8 cm, diameter x height) were aligned along the midline. All elements were filled with wood shavings, so that they looked identical. In all experimental phases, the elements were spaced 2.5 cm from one another, thus the overall length of the series was 54.5 cm. The position of the series of elements within the arena changed depending on the experimental conditions. During pre-training, training, re-training and sagittal test; the series was sagittally aligned with respect to the openings and symmetrically placed in the center of the apparatus (Fig 1A). The first element was 26.0 cm from the closest opening, which was the chick’s starting point; with the elements at 52.8 cm from the left and right walls. During fronto-parallel tests, the elements were displaced from left to right with respect to the opening. Thus, the new test series was rotated by 90° when compared with the training series (Fig 1B). In this experimental phase, only one opening was used with the series located 80 cm away from that opening.

In each experimental phase, from trial to trial, the apparatus was randomly rotated in the room to avoid potential use of external cues, such as distance from walls, windows, furniture etc.

## Experimental phases

### Pre-training

In the morning of the fourth day, chicks underwent the pre-training. Each bird was initially placed in the starting box, for about three seconds, and then by sliding the partition, it was allowed to enter the apparatus, where it was free to move around and become acquainted with the novel environment. As soon as the bird stopped to emit distress calls, the pre-training started. A mealworm piece was placed on top of the fourth element, thus it was not covered by wood-shavings and remained visible to the chick. Once the chick had found and eaten the piece of mealworm, a new piece was positioned on each trial and, on different trials, progressively buried in the wood-shavings, until it was completely concealed, requiring the bird to search for it by pecking on the container to retrieve the mealworm. This way, only chicks’ pecking responses to the fourth element, also labelled as ‘target element’ were reinforced. Once the mealworm was completely buried, only search on one element was allowed in each trial (i.e., after searching in an incorrect position the chick was removed from the arena and could not redirect towards another position or access any reward). The learning criterion to access progression to the next experimental phases was having located the correct position in three consecutive trials.

### Training

Training began two hours after pre-training. All the elements were uniformly filled with wood shavings and only the fourth element concealed a piece of mealworm.

At the beginning of each trial, the chick was placed in the starting box. After three seconds, the removable partition was lifted from above, letting the chick enter the apparatus. On each trial, only pecks at one element were allowed. A trial was over once the bird had pecked at any one element. A trial was considered correct when the chick pecked at the fourth element. A trial was considered null and thus terminated after 180 seconds in the absence of a response. The learning criterion consisted in at least eight correct responses over 20 valid trials [34, 37, 54]. All chicks reached the criterion and progressed to the subsequent phase.

### Retraining

Before each test, birds individually took part in a retraining. The experimental procedure was similar to that used during training. The retraining criterion consisted of three consecutive correct responses. All chicks reached such criterion in 5–10 minutes, and a few minutes after, they underwent the test.

### Sagittal test

Two hours after the training was over, each chick underwent the retraining and, immediately after, the sagittal test. This consisted of 20 consecutive trials and was conducted binocularly. On each trial, the chick was allowed to peck at a single element. A trial was over once the bird had pecked at one element.

Upon emitting a correct response, the chick may access a food reward. In fact, a piece of mealworm was present in the fourth element only on some pre-established trials (trial numbers 4, 5, 7, 10, 13, 14, 16 and 19), and chicks on those trials could gain the reward by correctly pecking at the target element. All other trials were unrewarded. We used the same reward schedule also for the fronto-parallel tests. This reward schedule prevented the extinction of responses over multiple unrewarded trials, and was preferred to a fully rewarded schedule which may have led to higher rates of learning during the test itself [34, 36, 55].

If no response occurred within 60 seconds, the trial was considered over. During this test, and the fronto-parallel tests, subjects’ behavior was observed from a screen connected to a video camera so as not to disturb the animals by direct observation. All trials were video-recorded and scored offline. At the end of each trial, the chick was gently relocated in the starting box and after approximately five seconds, it underwent a new trial.

### Fronto-parallel tests

In the morning of the fifth day, each bird took part in two fronto-parallel tests. Before each test, chicks underwent retraining and the interval between the first and the second fronto-parallel test was of two hours.

Monocular testing was conducted using temporary eye patches made of a removable conical-shaped patch of paper tape to occlude vision in a single eye, without preventing normal blinking. The patch was applied twenty minutes before the beginning of a monocular test to allow the chicks to adapt to their new condition of vision before being tested.

## Results

### Analysis

For each test, we considered three dependent variables: the accuracy (%), the Response times and the Direction of approach to the series.

#### Accuracy

For each trial, we scored the first peck emitted by each chick to any of the 10 elements and at the end of the 20 trials we computed the percentages (*number of pecks to a given element/20 x 100*), separately for each element.

#### Response times

We measured the time in seconds taken by each chick on every trial to peck any element, once the chick walked out of the starting box. We separately averaged correct and incorrect choices depending on the side selected: left (L), from the first left to the fifth left element or right (R), from the first right to the fifth right element. Response times were separately considered for Left Correct, Left Incorrect, Right Correct and Right Incorrect choices.

#### Direction of approach

In each trial, we tracked the trajectories of each chick approaching the series to determine if chicks walked toward the chosen element along a central or lateral path. In the sagittal test, we considered the path as ‘central’ when chicks walked along the series within 10 cm of either side of the series. Whenever the path was more than 10 cm over this virtual central corridor, the path was considered either left or right, depending on a chick’s walking direction, as shown in Fig 1A. In the fronto-parallel tests, we considered as central an approach to the series that occurred between the fourth left and the fourth right element, thus comprising both the fourth and the fifth elements. We considered the approach as left, if chicks approached the series starting from its more left lateral elements, 1L, 2L and 3L, and as right if the chicks approached the series corresponding to the three rightmost elements, 1R, 2R and 3R, as shown in Fig 1B.

We conducted the analyses on the 20 testing trials, then we restricted the analysis to the first four test trials. In fact the first trial rewarded in all tests was the 4^th^ trial, restricting the analyses allowed to exclude any effect of learning during testing. We conducted Bayes factor analyses using the default parameter values and JASP 0.11.1. We used the classification by Lee and Wagenmakers, 2013 to interpret the Bayes factor (BF).

### Sagittal test

#### Accuracy

A Bayesian Repeated Measures ANOVA with elements 1 to 10 as a repeated measures factor and the test order as a between factor showed a null evidence for the test order, BF = 0.189 and an extreme evidence for the selection of the elements, BF>100. Bayesian One Sample Test provided an extreme evidence for the selection of the fourth element (mean = 44.167, SE = 2.720; BF>100), and null evidence for the selection of all other elements; with the exception of the first and second elements for which the evidence was anecdotal (1^st^: mean = 15.417, SE = 2.674; BF = 2.350; 2^nd^: mean = 12.917, SE = 1.409; BF = 2.528). The Bayesian Factors, confirmed these outcomes also if the analyses were restricted to the first four trials (test order BF<0.001; selection of the elements, BF>100). An extreme evidence was provided for the selection of the fourth element (mean = 43.750, SE = 4.251; BF>100), and null evidence for the selection of all other elements.

#### Response times

A Bayesian Paired Samples t-Test showed a null evidence for response times for correct and incorrect responses (Correct: mean = 12.862, ES = 1.428; Incorrect: mean = 11.571, ES = 1.239; BF = 0.322). Null evidence in response times between correct and incorrect responses was reported also when this analysis was restricted to the first four trials (Correct: mean = 16.336, ES = 4.187; Incorrect: mean = 11.794, ES = 1.311; BF = 0.337).

We also analyzed the differences in response times in the first approach between left and right. Null evidence was provided when we analyzed the 20 testing trials (left approach: mean = 27.442, ES = 6.460; right approach: mean = 27.121, ES = 4.056; BF = 0.280), or the first four testing trials (left approach: mean = 18.133, ES = 4.275; right approach: mean = 18.117, ES = 2.538; BF = 0.509).

#### Direction of approach

A Bayesian Repeated Measures ANOVA with the direction of approach (central, left, right) as a repeated measure factors and the test order as between factor showed null evidence for the test order, BF = 0.331 and anecdotal evidence for the approach, BF = 1.773. We did not find any evidence of the preferential use of a direction of approach, when it was confronted with a chance expectation of 33.333 (central: mean = 37.917, ES = 4.014; BF = 0.658; left: mean = 38.958, ES = 5.710, BF = 0.545; right: mean = 23.125, ES = 4.103; BF = 0.071). When the analyses were conducted on the first four trials, the Bayesian Repeated Measures ANOVA showed null evidence for the test order, BF = 0.267 and for the approach, BF = 0.353. Null evidence was found for the direction of approach, when it was confronted with the chance expectation of 33.333 (left: mean = 33.333, ES = 7.624; BF = 0.215; central: mean = 41.667, ES = 6.324, BF = 0.823; right: mean = 25.000, ES = 6.730; BF = 0.106).

To explore if the direction of the first approach could affect accuracy, we conducted a Bayesian Repeated Measures ANOVA on the correct choices emitted when the series was approached from the left, centrally or from the right. Bayesian Factor provided null evidence for the direction of approach (left: mean = 18.958, ES = 2.657; central: mean = 11.667, ES = 2.618; right: mean = 13.542, ES = 2.052; BF = 0.758). These outcomes were confirmed also when the analyses were restricted to the first four trials (left: mean = 15.625, ES = 4.948; central: mean = 14.583, ES = 3.337; right: mean = 13.542, ES = 4.509; BF = 0.123).

### Left monocular fronto-parallel test

#### Accuracy

**Fig 2:**
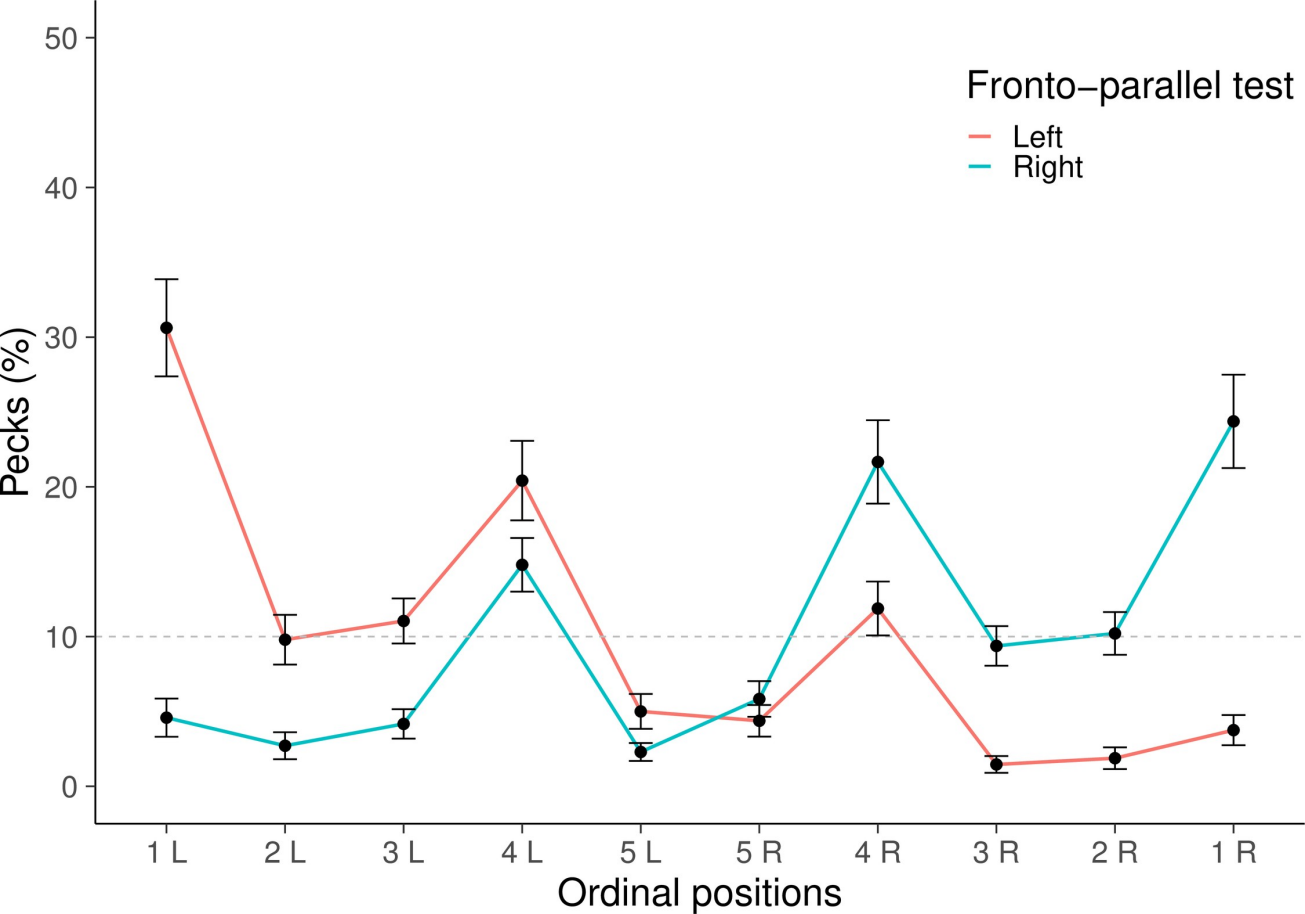
Accuracy in the monocular fronto-parallel tests. The graph represents the mean percentage + SE of choices for each position; the dotted line represents chance level (y = 10). Results of the left monocular fronto-parallel test, in red; results of the right monocular fronto-parallel test, in blue.

A Bayesian Repeated Measures ANOVA with elements as a repeated measure factors and the test order as between factor showed a null evidence for the test order, BF = 0.186 and an extreme evidence for the selected elements, BF>100. A Bayesian post hoc test provided moderate evidence for the selection of the fourth left element, 4L, over the fourth right one, 4R, BF = 7.570. A Bayesian One Sample Test provided very strong evidence for the selection of 4L (mean = 20.417, SE = 2.657; BF = 97.816), an extreme evidence for the selection 1L (mean = 30.625, SE = 1.801; BF>100). Null evidence was provided for the selection of all other elements including 4R (mean = 11.875, SE = 3.246; BF = 0.582; Fig 2). When the analyses were limited on the first four trials, the Bayesian Repeated Measures ANOVA provided a null evidence for the test order, BF<0.001 and a strong evidence for the selected elements, BF = 21.900. A Bayesian post hoc test provided moderate evidence for the selection of the fourth left element, 4L, over the fourth right one, 4R, BF = 7.110. A Bayesian One Sample Test provided a strong evidence for the selection of 4L (mean = 21.875, SE = 4.069; BF = 11.956), and a very strong evidence for the selection 1L (mean = 31.250, SE = 6.066; BF = 39.760). Null evidence was provided for the selection of all other elements including 4R (mean = 8.333, SE = 3.251; BF = 0.011).

#### Response times

**Fig 3:**
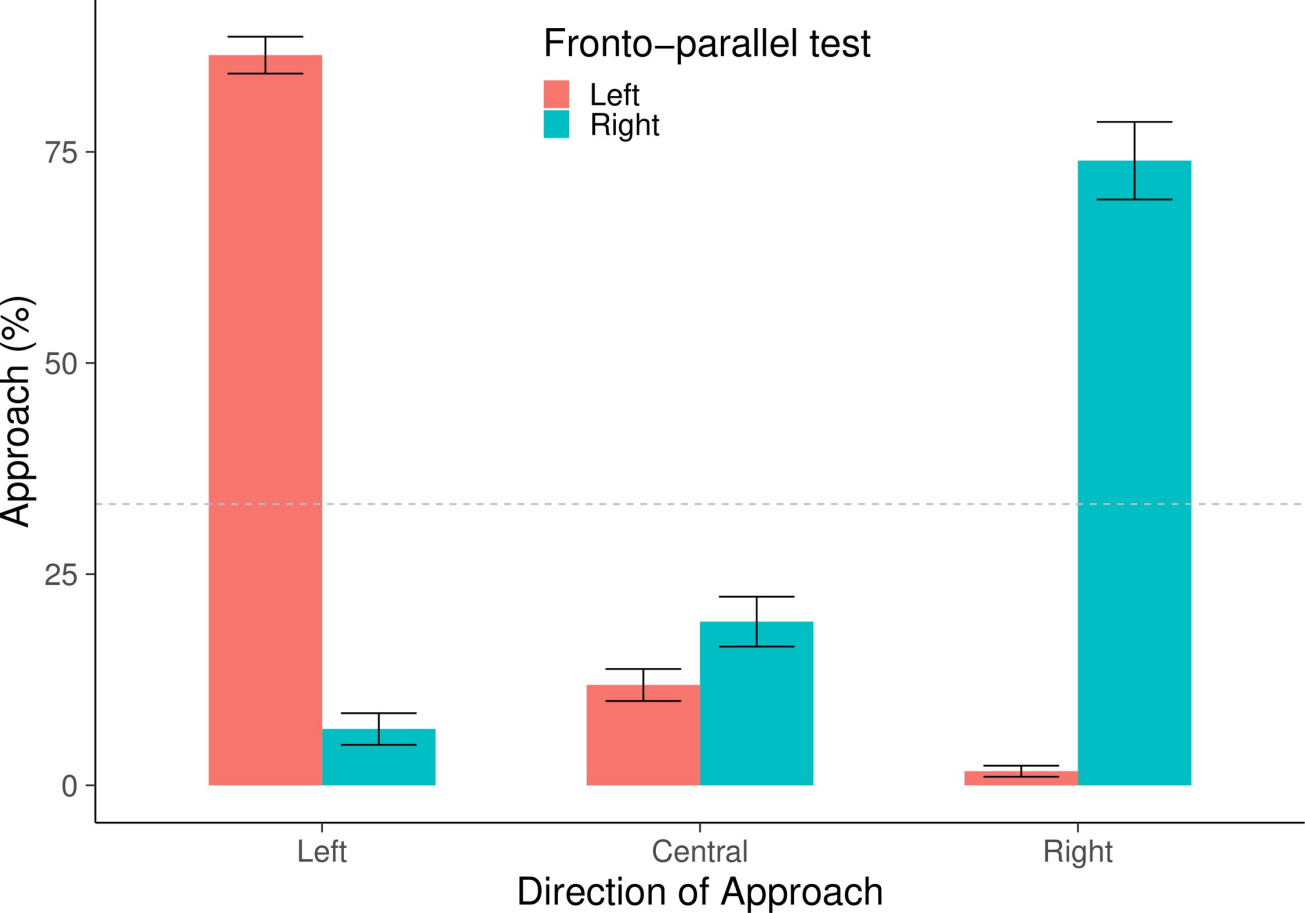
Response times in the monocular fronto-parallel tests. The graphs represent the mean percentage + SE of Response Times for correct and incorrect choices on either side. Results of the left monocular fronto-parallel test, in red; results of the right monocular fronto-parallel test, in blue.

A Bayesian Repeated Measures ANOVA showed a null evidence for the test order, BF = 0.362 and for the correct or incorrect responses on either side (Left Correct: mean = 36.894, ES = 8.653; Right Correct: mean = 32.256, ES = 6.138; Left Incorrect: mean = 20.582, ES = 2.074; Right Incorrect: mean = 26.196, ES = 3.141; BF = 0.359; Fig 3). When the analyses were limited on the first four trials, since the left-right response frequencies varied greatly, we analyzed each direction separately. Null evidence were reported for differences for correct and incorrect responses on the left as well as on the right (Left Correct: mean = 47.162, ES = 10.233; Left Incorrect: mean = 34.346, ES = 4.881; BF = 0.868; Right Correct: mean = 29.383, ES = 2.882; Right Incorrect: mean = 36.471, ES = 7.351; BF = 0.730).

Null evidence was provided also when we compared the response times in relation to the side (left vs. right) first approach to the series (Left: mean = 23.996, ES = 2.192; Right: mean = 24.440, ES = 7.956; BF = 0.482). Since in the first four trials no chicks approached the series from the right, we could not run the analysis on the very first trials (this indicates that also chicks that selected the right elements, did approach the series from the left side).

#### Direction of approach

**Fig 4:**
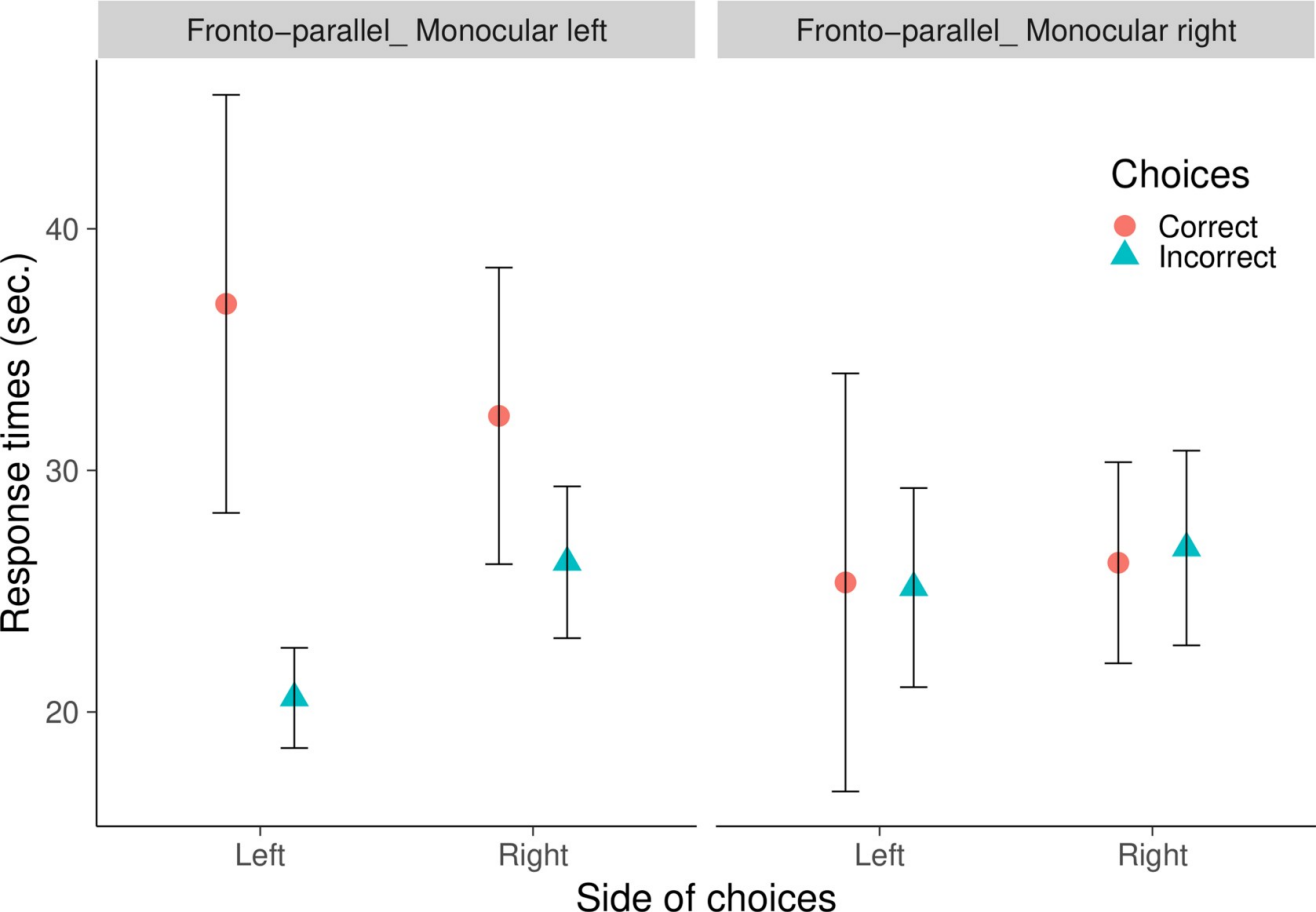
Direction of approach in the monocular fronto-parallel tests. The graphs represent the mean percentage + SE of direction of approach for left, central and right choices, respectively. Results of the left monocular fronto-parallel test, in red; results of the right monocular fronto-parallel test, in blue.

A Bayesian Repeated Measures ANOVA, with the direction of approach (central, left, right) as a repeated measures factor and the test order as between factor, showed a null evidence for the test order, BF = 0.274 and an extreme evidence for the direction of approach, BF>100 (Fig 4).

When each direction of approach was confronted with the chance expectation of 33.333, a Bayesian One Sample Test provided an extreme evidence for the selection of the left approach (left: mean = 86.458, ES = 2.180, BF>100) and null evidence for the central (mean = 11.875, ES = 1.899; BF = BF = 0.009) or for the right approach (mean = 1.667, ES = 0.650; BF = 0.002). When the analyses were conducted on the first four trials, the Bayesian Repeated Measures ANOVA showed null evidence for the test order, BF = 0.279 and extreme evidence for the approach, BF>100. The Bayesian One Sample Test provided an extreme evidence for the selection of the left approach (left: mean = 88.542, ES = 3.680, BF>100) and null evidence for the central (mean = 11.458, ES = 3.680; BF = BF = 0.020) and for the right approach (mean = 0.000, ES = 0.000).

To deepen how the direction of the first approach affected correct choices, we conducted a Bayesian Repeated Measures ANOVA on the correct choices emitted when the series was approached from the left, centrally or from the right. Bayesian Factor provided an extreme evidence for the direction of approach (left: mean = 25.208, ES = 2.941; central: mean = 0.833, ES = 0.491; right: mean = 5.208, ES = 1.184; BF>100). Post hoc comparisons revealed that the correct choices were emitted more when the series was approached from the left rather than centrally (BF>100) or from the right (BF>100). These outcomes were confirmed also when the analyses were restricted to the first four trials (left: mean = 27.083, ES = 5.812; central: mean = 3.261, ES = 1.795; right: mean = 0.000, ES = 0.000; BF>100). Post hoc comparisons revealed that the correct choices were emitted more often when the series was approached from the left rather than centrally (BF = 25.649) or from the right (BF>100).

### Right monocular fronto-parallel test

#### Accuracy

A Bayesian Repeated Measures ANOVA with elements as a repeated measure factors and the test order as between factor showed a null evidence for the test order, BF = 0.187 and an extreme evidence for the selected element, BF>100. A Bayesian post hoc test provided null evidence for the selection of 4R over 4L, BF = 0.933. A Bayesian One Sample Test provided an extreme evidence for the selection of 4R (mean = 21.667, SE = 2.786; BF>100), and for the selection of 1R (mean = 24.375, SE = 3.118; BF>100), and null evidence for the selection of all other elements. The evidence for the selection of 4L was moderate (mean = 14.792, SE = 1.793; BF = 7.427; Fig 3). When these analyses were conducted on the first four trials, the Bayesian Repeated Measures ANOVA showed a null evidence for the test order, BF<0.001 and strong evidence for the selected element, BF = 20.366. A Bayesian post hoc test provided an anecdotal evidence for the selection of 4R over 4L, BF = 1.109. A Bayesian One Sample Test provided an extreme evidence for the selection of 4R (mean = 25.000, SE = 3.686; BF>100), and a strong evidence for the selection of 1R (mean = 25.000, SE = 4.991; BF = 14.225), and null evidence for the selection of all other elements, including the 4L element (mean = 13.542, SE = 3.680; BF = 0.531).

#### Response times

A Bayesian Repeated Measures ANOVA showed a null evidence for the test order, BF = 0.346 as well as for the correct or incorrect responses on either side (Left Correct: mean = 25.362, ES = 3.537; Right Correct: mean = 26.177, ES = 4.162; Left Incorrect: mean = 25.149, ES = 4.120; Right Incorrect: mean = 26.787, ES = 4.030; BF = 0.070; Fig 4). Anecdotal evidence was provided also when the analysis of correct and incorrect response times was limited to the first four trials (Left Correct: mean = 48.728, ES = 10.777; Right Correct: mean = 31.262, ES = 4.774; Left Incorrect: mean = 36.742, ES = 10.120; Right Incorrect: mean = 37.990, ES = 5.534; BF = 1.162).

When we tested for any effect on response times depending on the left vs. right side of the first approach, Bayesian Factors provided null evidence when considering 20 testing trials (Left: mean = 27.442, ES = 6.460; Right: mean = 27.121, ES = 4.056; BF = 0.280), and anecdotal evidence when the analysis was conducted on the first four testing trials (Left: mean = 57.110, ES = 8.756; Right: mean = 37.413, ES = 4.504; BF = 1.358).

#### Direction of approach

A Bayesian Repeated Measures ANOVA the direction of approach (central, left or right) as a repeated measures factor and the test order as between factor showed a null evidence for the test order, BF = 0.275 and extreme evidence for the direction of approach, BF>100. When each direction of approach was confronted with the chance expectation of 33.333, a Bayesian One Sample Test provided an extreme evidence for the selection of the right approach (mean = 73.958, ES = 4.594, BF>100) and null evidence for the central (mean = 19.375, ES = 2.954; BF = 0.050) or for the left approach (mean = 6.667, ES = 1.871; BF = 0.007; Fig 4). When the analyses were conducted on the first four trials, the Bayesian Repeated Measures ANOVA showed null evidence for the test order, BF = 0.275 and extreme evidence for the approach, BF>100. The Bayesian One Sample Test provided an extreme evidence for the selection of the right approach (mean = 59.375, ES = 5.172, BF>100) and null evidence for the central (mean = 33.333, ES = 4.430; BF = 0.215) or for the left approach (mean = 7.292, ES = 3.185; BF = 0.014).

To explore if the direction of the first approach can affect correct choices, we conducted a Bayesian Repeated Measures ANOVA on the percentage of correct choices emitted when the series was approached from the left, centrally or from the right. Bayesian Factors provided an extreme evidence for the direction of approach (left: mean = 2.917, ES = 1.039; central: mean = 10.000, ES = 1.880; right: mean = 23.542, ES = 2.527; BF>100). Post hoc comparisons revealed that the correct choices were emitted more frequently when the series was approached from the right than centrally (BF = 38.422) or the left (BF>100). When the analyses were conducted on the first four trials, Bayesian Factors showed a strong effect for the direction of the first approach (left: mean = 3.125, ES = 1.724; central: mean = 18.750, ES = 4.322; right: mean = 16.667, ES = 3.885; BF = 12.486). Post hoc comparisons revealed that the correct choices were emitted more often when the series was approached from the right than from the left (BF = 10.282); no difference emerged comparing the right vs. central approach (BF = 0.224).

## Conclusions

The present study aims at disentangling the engagement of either hemisphere in determining the spatial bias as highlighted in the literature on numerical ordinal processing. We employed three different measures aimed to unveil how the spatial bias is connected with the processing conducted by each hemisphere. Compared to previous studies, we employed two new measures: one focused on the direction of choices, Direction of approach, and one focused on response velocity, response times.

Results of the sagittal test performed in binocular conditions of vision showed that day-old chicks correctly identified the target element, i.e., the fourth container. This evidence supports the outcomes of previous studies [24, 36–38] and sustains the idea that birds are precociously able to extract a spatial-ordinal rule to locate a food source. Accuracy data, on either the whole test or the very first four (i.e., unrewarded) trials, revealed that the birds showed higher proportion of choices of the target element over all other elements. The direction of approach did not show any lateral bias, indicating that neither hemisphere is dominant in this task. Again, data of the whole test as well as of the unrewarded very first trials were alike. A possible explanation could be that chicks, in identifying the target element in a sagittal series of fixed and equidistant elements, used both spatial and numerical information [36]. Response times neither showed any speed difference between correct and incorrect responses, considering either the whole test or the very first trials. Results from the monocular fronto-parallel test showed that chicks could generalize the learnt rule to a new series characterized by a different orientation with respect to the one they experienced during training. This evidence is in line with the existing literature, supporting a precocious use of spatial-ordinal information in new-born domestic chicks [24, 36, 38].

In monocular fronto-parallel tests, the birds confined their responses to the visible hemifield. Reliance on the clear hemifield, corresponding to the seeing eye, has been also reported in domestic chicks in tasks requiring processing of non-spatial information [56]. In such a restricted condition of vision, chicks could only see the side of the series ipsilateral to their unobscured eye. Consequently, only one end of the series was clearly visible and it was used by chicks as a benchmark to start to ‘count’. This behavior was supposed to be based on the condition of vision, which probably has driven the animals to start their scanning of the series from the first element—either on the left- or right- end—onto which they anchor their proto-counting [36, 37]. The direction of choices, aimed at investigating this aspect, showed chicks approached the series from their unobscured side. In a left monocular condition, chicks approached the series starting from their left, and in a right monocular condition, the birds approached the series from their right. The hypothesis that a benchmark might be necessary to anchor an element among the others to start to count is supported by the fact that in both fronto-parallel tests, behind the fourth, the only other selected element was the first one. Up to now it was unclear why animals, rats [57, 58], chicks [36, 37] and fish [59] respond consistently at the first element in a spatial-ordinal task. It has been suggested that this behavior is perhaps related to the perceptual salience of the first element in a series: it is the first element the animal encounters while approaching the series, and such element is identical to the target element in the correct position [59]. Both hemispheres seem therefore to exploit the same strategy to start to scan the series from the most lateral element in the readily visible side of the space. This suggests that both hemispheres use a spatial cue to perform a proto-counting. In fronto-parallel tests, there was no effect in response times. In each condition of vision, the response times of either correct or incorrect choices on either side were equal. This indicates that in this paradigm the direction of choice and not the speed is the variable to assess the SNA.

An insightful reflection perhaps arises by comparing the behavioral responses in the fronto-parallel test performed under monocular conditions. Even if the side bias depended on the eye in use, in both conditions chicks showed a lateral bias. This supports the idea that numerical performance is strictly intertwined with space. This opens a parallelism with humans. Although the specific orientation of the SNA varies with the influence of cultural conventions, its presence across all cultures suggests that the spatial-numerical association is a universal cognitive strategy [60]. Comparative studies on numerical processing suggest that numerical knowledge constitutes a domain-specific cognitive ability [54, 61–65], with a dedicated neural substrate located in the parietal cortices [66–68]. In 2015, neurons selective to numbers were found in a brain association area (*nidopallium caudolaterale*, NCL) of crows [69]. A model that assumes differential encoding, processing and integration by the two hemispheres for spatial and numerical information followed by a subsequent integration of both information has been recently proposed as an explanation of the SNA [34]. Even if here we did not test chicks in binocular condition of vision, previous studies demonstrated how chicks tested in binocular and in left monocular condition of vision behaved alike [34, 36]. This allows inferring that whenever both eyes, and consequently both hemispheres, are processing a spatial-ordinal task, chicks would show a behaviour similar to the one shown by left monocular chicks. From this perspective, the left bias would occur because of an availability of spatial information, which in the chick forebrain is processed by the right hemisphere [70–76]. Whenever the two hemispheres are involved in a spatial-ordinal task, the availability of the spatial information, coherent with the numerical one, would result in the right hemisphere that takes control of the birds’ behavior. This would favor a preferential allocation of attention leftward, inducing a bias to ‘count’ from left to right.

In summary, these data support the idea that young and mostly inexperienced animals show a SNA. The new measures assessed in this study indicate that SNA can be highlighted in this behavioral paradigm by analyzing the direction of choice, whereas response times are not a relevant measure. This research provides some insight on how to investigate the origin of the association between numbers and space, stressing the relevance of selecting the appropriate behavioral measures.

## Supporting information

S1 Video An illustrative video of the fronto-parallel test. (MP4)
